# Annotations

**Published:** 1893-04-29

**Authors:** 


### Annotations.

**Mr. Balfour and "Research."** One of the "Books of the Week" is Mr. A. J. Balfour's *Essays and Addresses*. Mr. Balfour is, perhaps, the most interesting personality, after Mr. Gladstone, of all the members of the House of Commons. He is a man of science devoted to non-scientific pursuits. One of the *Addresses* is that on "Progress," delivered in his capacity of Lord Rector to the students of the University of Glasgow. In that address Mr. Balfour said, among other things, that "Our senses are very few and very imperfect. They were, unfortunately, not evolved for purposes of research." This dictum proved displeasing to Sir Edward Reed at the time it was delivered, and now that it is published in a permanent form it appears to be equally distasteful to the *Times* reviewer. The main quarrel with Mr. Balfour seems to be that he dares to think that our senses were "not evolved for purposes of research." But this, surely, is obvious? Mr. Balfour does not mean that our senses are incapable of research, but that the chief use of our senses is not "research." And this is manifest on a very cursory glance of the leading occupations of mankind. Let us take mankind in the mass, as philosophers do and must. What proportion of men are given to anything which can properly be called "research"? One in a hundred thousand perhaps, even if this be not a much too liberal percentage. If our senses had been "evolved for research" every normal man in the hundred thousand would have been a researcher, just as every normal man sees, feels, eats, digests, runs, walks, and so on. Research is an incident in human experience, as war is. A very important incident we grant, and an incident which is necessary to progress. But when all is said, the great world knows little of it, and profits little by it; the more is the pity. The researcher must always be more or less like John the Baptist—a voice crying in the wilderness. All honour to him that in spite of his isolation and the neglect to which he is subjected, he still continues his protests to an unheeding generation and his labours on their behalf. Philosophically Mr. Balfour spoke strict fact when he said our senses were not evolved for research, though we may be entitled to feel very dissatisfied when we admit the general truth of the proposition.

**Laziness as a Cure.** A young lady made pathetic complaint in last week's *Spectator* that everybody extols education, whilst nobody takes the trouble to utter even the very mildest protest on the other side. She herself takes up cudgels for the unrepresented defence, and, having had, what she considers the disadvantage of too much education, proceeds to show by many amusing and some serious facts how very considerably she has suffered and continues to suffer in consequence. Similarly the world is mad upon work, and poor Laziness cannot so much as get a hearing. This is not a happy generation; and one of the chief reasons is that it does not know how to be lazy. It cannot write a sentence at a sitting: it must write a whole essay. It cannot be content with five hundred a year, but kills itself in the too often futile endeavour to make five thousand. But the sentence might sometimes be better than the essay, and five hundred a year would make some people happier than five thousand. Judicious laziness is fine as a prophylactic, and still more excellent as a cure. The brain of the literary man makes one constant plea to him. It says, work me as hard as you will for a limited period, but give me some rest, give me some play, let me lie fallow in the fallow season. If Shakespeare had lived in our day we should have had no "Henry VIII." and no "Hamlet." Instead, he would have written articles for the weeklies, unless, indeed, the weeklies had refused his articles as not quite conventional enough for their pages. Considering the enormous number of living writers, it is wonderful how little is produced which promises to attain to immortality. One reason is because we are never lazy. Nobody knows so well as doctors the deadly consequences of the "eternal grind"; but nobody grinds so eternally. We pay for it; but our example is no warning. The mortality among working doctors is just about double that of working clergymen. Yet doctors "grind on" more strenuously than before. Is life worth living if we cannot extract a little wholesome satisfaction out of it; a little careless ease; a little genial freedom and world-abandoning laziness? Hardly!

**The Afternoon Nap.** It is a solemn subject, this of the afternoon nap. *Wit and Wisdom* has collected twenty-four "eminent" opinions upon it. Probably not one person in a hundred who reads the opinions will be enabled thereby to decide whether he ought to take an afternoon nap or not, but he will discover that brain-workers as a class have a great faculty for pleasing themselves in this matter, and find their account in so doing. Life, after all, is not made for physiology, but physiology for life. In other words, Nature, apparently, never intended the doctor to be the chief hierarch of well humanity, but only a helper and a nurse of humanity when it is sick. But since it is now our *metier* to improve upon Nature, the doctor is beginning to assert himself in a new capacity. He claims to be something more than a helper and a nurse in sickness. He professes to be a teacher and a scientific prophet for the well. In this latter capacity he has very decided opinions about the afternoon nap. For the healthy the nap in the afternoon is not necessary, and the brain will not demand it. If a man finds himself napping at that time, either he has eaten too much at his mid-day meal, or his cerebral circulation is feeble. It is the universal habit of the pig that is being fattened to sleep in his sty after his mid-day meal. The working horse, on the other hand, which gets nothing at midday but a feed of corn—a small but highly nutritious meal—does not sleep at all after it, but is fresh and ready for work in half-an-hour. One would like to persuade all literary-workers to work in the day and to sleep in the night. They should never go for more than eight hours a day. Early rising would be good for most of them. A cup of coffee and a piece of toast at half-past six might be followed by an hour's work from seven to eight. The whole hour between eight and nine should be devoted to a thoroughly good breakfast and a short walk. Work from nine till twelve. Half-an-hour should then be spent in gentle sauntering in the fresh air, and a light lunch should follow—say a chop and bread, with a modicum of light pudding, accompanied by a small glass of lager beer. From one to two a pipe and a saunter, and at two a cup of black coffee. From two to four, work; at four a cup of afternoon tea, and a rest until five. From five to six or half-past, work; and at half-past six the real labours of the day should be over and completed. At seven, a good, well-cooked, appetising, slowly eaten dinner, followed by one cup of black coffee, but no tea. At a quarter to eleven a small cup of cocoa and one or two pieces of toast. At eleven, bed, and sleep until six or six thirty. The brain-worker should not work more than five days a week in this fashion. He should have two days of leisure in the week. The first of these should be devoted to brisk and thoroughly fatiguing exercise in the open air, and the second to lolling, lounging, a little light reading, and the like. This is the kind of life which physiology would suggest for the brain-worker, and in this "scheme of life" there is neither place nor necessity for the afternoon nap.

